# OPTIMIZING INDIVIDUALS’ DAILY FUNCTION: A BOOLEAN NETWORK APPROACH FOR IDENTIFYING INTERVENTION SEQUENCES

**DOI:** 10.1093/geroni/igz038.1379

**Published:** 2019-11-08

**Authors:** Xiao Yang, Xiao Yang, Nilam Ram, David Conroy, Aaron Pincus, Denis Gerstorf

**Affiliations:** 1 Pennsylvania State University, University Park, Pennsylvania, United States; 2 Humboldt-Universität zu Berlin, Berlin, Berlin, Germany

## Abstract

Development and aging are the product of a process wherein an individuals’ functional components co-act to produce change. System dynamics can be described using a variety of methods. In this paper we illustrate how Boolean network methods may be used to describe the sequences of emotion and behavior states that lead to a stable equilibrium – e.g., healthy function; and the interventions needed to push an individual toward healthier equilibria. We applied Boolean network models to intensive longitudinal data obtained from 150 participants (age 18-89 years) to describe individuals’ on-going psychosocial dynamics and identify the specific social behaviors that may be driving them toward undesirable and/or desirable equilibria (e.g., high and low negative emotions). Results are discussed with respect to how they inform theory about developmental systems, and construction of interventions meant to guide individuals toward healthy aging.

